# Bio-mimicking nano and micro-structured surface fabrication for antibacterial properties in medical implants

**DOI:** 10.1186/s12951-017-0306-1

**Published:** 2017-10-02

**Authors:** Alka Jaggessar, Hesam Shahali, Asha Mathew, Prasad K. D. V. Yarlagadda

**Affiliations:** 1 Science and Engineering Faculty, Queensland University of Technology, Brisbane, Australia; 2 Institute of Health and Biomedical Innovation, Queensland University of Technology, Brisbane, Australia

**Keywords:** Nanofabrication, Bio-mimicking, Medical implants, Bactericidal mechanisms, Superhydrophobicity, Antibacterial behaviour

## Abstract

Orthopaedic and dental implants have become a staple of the medical industry and with an ageing population and growing culture for active lifestyles, this trend is forecast to continue. In accordance with the increased demand for implants, failure rates, particularly those caused by bacterial infection, need to be reduced. The past two decades have led to developments in antibiotics and antibacterial coatings to reduce revision surgery and death rates caused by infection. The limited effectiveness of these approaches has spurred research into nano-textured surfaces, designed to mimic the bactericidal properties of some animal, plant and insect species, and their topographical features. This review discusses the surface structures of cicada, dragonfly and butterfly wings, shark skin, gecko feet, taro and lotus leaves, emphasising the relationship between nano-structures and high surface contact angles on self-cleaning and bactericidal properties. Comparison of these surfaces shows large variations in structure dimension and configuration, indicating that there is no one particular surface structure that exhibits bactericidal behaviour against all types of microorganisms. Recent bio-mimicking fabrication methods are explored, finding hydrothermal synthesis to be the most commonly used technique, due to its environmentally friendly nature and relative simplicity compared to other methods. In addition, current proposed bactericidal mechanisms between bacteria cells and nano-textured surfaces are presented and discussed. These models could be improved by including additional parameters such as biological cell membrane properties, adhesion forces, bacteria dynamics and nano-structure mechanical properties. This paper lastly reviews the mechanical stability and cytotoxicity of micro and nano-structures and materials. While the future of nano-biomaterials is promising, long-term effects of micro and nano-structures in the body must be established before nano-textures can be used on orthopaedic implant surfaces as way of inhibiting bacterial adhesion.

## Background

Orthopaedic implants carry out joint or bone function within the human body, include hip and knee replacements, plates, pins, rods and screws [1], and have an associated risk of bacterial infection post-surgery. Sources such as the implant itself, surgical tools, surgical theatre and contaminated disinfectants are potential bacteria carriers [2]. Implant materials are preferential sites for bacterial adhesion, compromising patient immunity and increasing risk of bacterial infection, leading to prolonged hospitalisation, long-term antibiotic therapy, bacterial resistance and the development of superbugs, revision surgery or death [3–5]. The number of revision and primary hip replacement surgeries grew by 50% in USA between 1993 and 2004, with an average cost of $31,000 per patient [6]. Similarly, the Australian Orthopaedic Association reported a steady increase of hip, knee and shoulder procedures from 1999 to 2016, with 23% of first revision surgeries required due to failure by bacterial infection [7].

Bacterial infection occurs through the formation of a self-produced polysaccharide matrix, known as the biofilm, which attaches to the surface of the implant and protects bacteria from pharmacological therapies [4]. Surface topography and roughness have great influence on the attachment of bacteria to a material surface and therefore, on biofilm formation. Factors dictating this attachment include hydrophobicity, electrostatic interactions, van der Waals forces, and steric hindrance [8].

Several studies have attempted to mimic the nano-texture of naturally occurring surfaces such as cicada and dragonfly wings, lotus leaves and shark skin [8–16]. The surfaces of cicada and dragonfly wings exhibit bactericidal properties towards some bacteria strains due to its nano-scale pillar structure [11, 17, 18]. The nano and micro-scale hierarchical structure on lotus leaves are responsible for its unique superhydrophobic and self-cleaning properties [19–22]. The large number of nano-scale spatula found on gecko feet allow it to support many times its body weight and adhere to various surfaces. The discovery of these structures and their various resulting properties has led to a large research focus in mimicking the surface structure of these naturally occurring surfaces to reproduce their behaviours.

Since 2006, researchers have focussed on the elimination of bacteria by the physical topography of material surfaces, rather than chemical mechanisms. Studies postulate that bacteria cell walls stretch and disfigure when they interact with textured surfaces. Stretching occurs in the regions between structures and if sufficient, cell rupture and death occur [11, 17]. Nano and micro-structures drastically increase contact adhesion area, creating more effective bactericidal properties than flat surfaces. Bactericidal efficiency of the surface is impacted by structure height, radius and spacing [23]. Surfaces that prevent bacterial adhesion are classified as either bactericidal or anti-biofouling surfaces. Anti-biofouling surfaces repel and prevent cell attachment due to surface chemistry or unfavourable surface topography, whereas bactericidal surfaces disrupt the cell, causing death [10]. This review article will explore various natural and fabricated nano-textured surfaces and their underlying physical properties aiding them to inhibit bacterial contamination. Bactericidal mechanisms and the mechanical stability of nano-structures are also discussed.

The prevalent use of orthopaedic implants has encouraged the development of biomaterials. However, there are inherent difficulties in replicating the behaviour of organic material such as bone, onto ‘non-living’ materials. Biomaterials must successfully function within the human system despite being a foreign material, must be biologically compatible and have appropriate mechanical, wear and corrosion properties [1]. Achieving the optimal combination of properties is often a trade-off. For example, whilst titanium has an elastic modulus similar to bone resulting in excellent osseointegration, its low static and fatigue strengths restrict its use in contacting joint surfaces which experience relative motion and high load bearing. Hence, the Ti-6Al-4V alloy is preferred to pure titanium in orthopaedic implants, as well as for its improved passivity and corrosion resistance [24, 25]. Stainless steel is also used in medical applications for its mechanical properties, corrosion resistance, ease of manufacturing and cost effectiveness. However, low biocompatibility and high elasticity modulus limit its use in implants. Although titanium alloys are less cost effective than stainless steel, its lightweight and biocompatibility properties make it favourable for implant applications [5, 26, 27]. Various coating methods, surface modification and implanting ions such as silver, calcium phosphate and hydroxyapatite improve bone regeneration, tissue response and antibacterial properties of the implant surface [25, 28]. These coatings however, tend to lose their effectiveness over time and may cause toxicity effects in the body [26, 29].

Like any foreign material, the introduction of implants into the body carries the inherent risk of bacterial infection [30]. Sources of infection can be present externally and/or internally, arising from the operating environment, surgical equipment and attire, patients’ skin, and pre-existing bacteria in the patient’s body. These bacteria [primarily *Pseudomonas aeruginosa* (*P. aeruginosa*)*, Staphylococcus aureus* (*S. aureus*) and *Staphylococcus epidermidis* (*S. epidermidis*)] adhere to the implant surface and form a periprosthetic biofilm layer, highly immune to antibacterial treatment. This infection may cause localised inflammation or may expand further into the body, inducing chronic infection. In either case, early implant replacement can prevent the possibility of amputation or death [30]. To reduce the need for revision surgery, researchers have put a large focus on developing materials with nano-structured surfaces to inhibit the growth of bacteria, biofilm formation and ultimately bacterial infection, without side effects.

## Nano-structures and natural surfaces

Natural surfaces provide ongoing and ever-increasing sources of inspiration and motivation for researchers to mimic their antibacterial behaviour [12]. Some natural surfaces decrease adherence and proliferation rates of algal spores, particles and bacteria, and are categorised as either anti-biofouling or bactericidal. Anti-biofouling surfaces (e.g. lotus leaves, taro leaves and shark skin) repel bacterial adhesion and cell attachment due to the presence of micro and nano superhydrophobic structures and surface patterns. Bactericidal surfaces, such as dragonfly and cicada wings and gecko skin, disturb and kill bacteria, with some surfaces exhibiting both anti-biofouling and bactericidal behaviour [11]. This section discusses various naturally occurring antibacterial surfaces and their nano-structures. Table 1 lists surface topographies of natural surfaces exhibiting antibacterial properties.

**Table 1:** Surface topography of natural surfaces exhibiting antibacterial properties

Natural surface	Surface	Species	Nano-texture	Geometry	Contact angle (°)	References
Plant	Taro leaf	*C. esculenta*	Polygon shape	Bulge: 15–30 μm diameter, Papilla: 10–15 μm diameter	159 ± 2	[31, 56]
	Lotus leaf	*N. nucifera*	Micro-size bulge shape	Bulge: 1–5 μm height	142 ± 8.6	[34, 57]
Animal	Gecko skin	*L. steindachneri*	Hair like nano-structure	4 µm length, top radius of 10–20 nm and submicron spacing	150	[42, 43]
	Shark skin	Spiny Dogfish	3D riblet micro-structure	Triangular riblets, 100–300 µm width, 15 µm peak radius, 200–500 nm height and 100–300 µm spacing	–	[37]
		*C. brachyurous*	3D riblet micro-structure	5 riblets 200–300 µm in height, 20–30 µm diameter and 50–80 µm riblet spacing	–	[38]
Insect	Cicada wing	*M. intermedia*	Nano-pillar (conical shape)	Height: 241 nm, diameter: 156 nm, spacing: 165 nm	135.5	[46]
		*A. spectabile*	Nano-pillar (conical shape)	Height: 182 nm, diameter: 207 nm, spacing: 251 nm	113.2	[46]
		*C. aguila*	Nano-pillar (conical shape)	Height: 182 nm, diameter: 159 nm, spacing: 187 nm	95.7	[46]
		*C. maculata*	Nano-pillar (conical shape)	Height: 309 nm, diameter: 97 nm, spacing: 92 nm	76.8 ± 13.9	[45]
		*P. scitula*	Nano-pillar (conical shape)	Height: 282 nm, diameter: 84 nm, spacing 84 nm	91.9 ± 5.9	[45]
		*M. hebes*	Nano-pillar (conical shape)	Height: 164 nm, diameter: 85 nm, spacing: 95 nm	78.4 ± 5	[45]
		*L. bifuscata*	Nano-pillar (conical shape)	Height: 200 nm, diameter: 90 nm, spacing: 117 nm	81.3 ± 8.3	[45, 58]
		*M. conica*	Nano-pillar (conical shape)	Height: 159 nm, diameter: 95 nm, spacing: 115 nm	93.9 ± 8.3	[45]
		*M. durga*	Nano-pillar (conical shape)	Height: 257 nm, diameter: 89 nm, spacing: 89 nm	134.8 ± 5.7	[45]
		*A. bindusara*	Nano-pillar (conical shape)	Height: 234 nm, diameter: 84 nm, spacing: 91 nm	135.5 ± 5.2	[45, 58]
		*M. mongolica*	Nano-pillar (conical shape)	Height: 417 nm, Diameter: 128 nm, Spacing: 47 nm	123.3 ± 12.7	[45]
		*P. radha*	Nano-pillar (conical shape)	Height: 288 nm, diameter: 137 nm, spacing: 44 nm	136.5 ± 5.2	[45]
		*D. vaginata*	Nano-pillar (conical shape)	Height: 363 nm, diameter: 132 nm, spacing: 56 nm	141.3 ± 3.3	[45]
		*D. rasingna*	Nano-pillar (conical shape)	Height: 316 nm, diameter: 128 nm, spacing: 47 nm	141.6 ± 4.5	[45]
		*M. opalifer*	Nano-pillar (conical shape)	Height: 418 nm, diameter: 148 nm, spacing: 48 nm	143.8 ± 6	[45, 58]
		*T. vacua*	Nano-pillar (conical shape)	Height: 446 nm, diameter: 141 nm, spacing: 44 nm	144.2 ± 6.8	[45]
		*T. jinpingensis*	Nano-pillar (conical shape)	Height: 391 nm, diameter: 141 nm, spacing: 46 nm	146 ± 2.6	[45]
		*C. atrata*	Nano-pillar (conical shape)	Height: 462 nm, diameter: 85 nm, spacing: 90 nm	137.9	[58]
		*P. claripennis*	Nano-pillar (conical shape)	Height: 200 nm, base diameter: 100 nm, cap diameter: 60 nm, spacing: 170 nm	147 ± 47	[11, 12]
	Dragonfly wing	*S. vulgatum*	Nano-pillar	Height: 80–90 nm, diameter: 150–20 nm	–	[49]
	Butterfly wing	*Blue M. didius*	Scales with aligned micro-grooves	Diameter: 1–2 µm, spacing: 1–2 µm	160	[59]

### Plant leaves

#### Taro leaves

Taro leaves (*Colocasia esculenta*) have anti-biofouling, hydrophobic and self-cleaning characteristics due to their well-ordered micro and nano-patterned surface [31]. The basic surface structure of taro leaves consists of micro-scale elliptical bumps (10–30 µm in diameter), which are covered by hierarchal, waxy nano-scale epicuticular crystals [21, 31]. The presence of these bumps increases the contact angle (90°–150°) of the surface, making it superhydrophobic in nature [31, 32]. As a result, dirt particles and bacteria preferentially attach to water droplets on the surface, instead of the surface itself. Dirt and contaminants then roll off the leaf with the water droplet, simultaneously cleaning the leaf [20, 31]. For this process to work, air must always be entrapped among the nano-structures, even under varying water conditions. This property is influenced by wettability and surface roughness. Nano-structures with highly dense patterns improve the reduction rate of bacteria and particle attachment under water, compared to low density patterns [31, 33].

#### Lotus leaves

Like taro leaves, the anti-biofouling and self-cleaning characteristics of lotus leaves (*Nelumbo nucifera*) has been the subject of intense research. The surface structure is similar to that of taro leaves, exhibiting a pattern of micro-scale elliptical bumps, covered by nano-scale crystals. This results in high contact angles, giving the surface its superhydrophobic nature. This in turn causes water droplets to roll off the surface of the leaf, gathering dirt particles and contamination [20].

Cheng et al. [34] demonstrated the self-cleaning effect of these micro and nano-structures, by comparing untreated lotus leaves with annealed lotus leaves. Annealing (150 °C for 1 h) eliminated all nano-crystals on the surface, while micro-structures (5–10 µm height) remained. The untreated lotus leaf had a higher contact angle (142.4° ± 8.6°) compared to the annealed leaf (126.3°), and the smooth wax surface had a contact angle of 74°. This shows that the presence of nano-structures does indeed increase the contact angle of the surface.

This study also suggests that the micro-scale bump pattern has a significant influence on hydrophobicity, as its presence increased the contact angle by 70%. The nano-crystals had less of an impact, increasing the hydrophobicity of the surface by 13% [34]. The resistance of taro and lotus leaves towards biological and non-biological particles is due to the physiochemical interaction between the cell and the surface roughness of the leaf. This behaviour has increased research interest in applications such as self-cleaning paint, clothes, windows, bio-repellent coatings and low friction surfaces [31].

#### Animal skin

##### Shark skin

The surface of shark skin has self-cleaning, anti-biofouling, hydrophobic, drag reducing and aerodynamic characteristics. The anti-biofouling and self-cleaning properties of shark skin is attributed to micro-structured riblets found on its dermal denticles. The size and shape of these denticles vary between shark species, as well as inhabited locations [35]. The micro-structure of the skin also facilitates high speed swimming (up to 90 km/h), allowing sharks to hunt their prey [36]. The presence of these micro-structures distinguish sharks from other aquatic species, such as whales, which are covered by barnacles [35].

Spiny Dogfish (mud) sharks have a skin surface comprising of triangular riblets, which have a width of 100–300 µm, peak radius of 15 µm, height of 200–500 nm and a 100–300 µm centre to centre spacing [37]. Copper shark (*Carcharhinus brachyurous*) skin is composed of placoid scales, with small grooves in the direction of water flow. Every scale on the Copper shark has five riblets 200–300 µm in length, 20–30 µm in height and 50–80 µm in width [38]. Although the ridges have smooth surfaces, nano-patterned projections are evident on the grooves [36].

Studies have shown that the presence of micro-riblets reduces friction caused by turbulent water flow by lowering drag and encouraging anisotropic flow, helping sharks to conserve energy and reach high swimming speeds [39]. Silicone patterned surfaces designed to mimic the micro-structure of shark skin has reduced drag resistance to submarines and ships by 15% and algae cell attachment by 67% [40].

##### Gecko skin

Gecko feet have strong adhesion properties and can selectively adhere to a variety of surfaces. This behaviour is due to the periodic array of hierarchal micro-scale keratinous hairs, known as setae. These hairs are approximately 30–130 µm in length, 5 µm in diameter and split into hundreds of nano-scale spatula, 200–500 nm in diameter [22]. Each spatula produces a small van der Waals force, which collectively creates large adhesion and anti-wetting properties [41, 42]. The hair like structures create a contact angle of 150° and produce bactericidal effects against certain gram-negative and gram-positive bacteria [42, 43]. Efforts have been made to replicate these nano-hairs using acrylic, which produced a surface that killed bacteria after 1 week of incubation. Artificially produced structures were less efficient at killing gram-positive *S. mutans* cells. This study found that gecko skin had an 88% success rate at killing gram-negative bacteria, compared to a 66% rate against gram-positive bacteria [43]. The resistance of gram-positive bacteria is most likely due to the higher stiffness and thickness of the cell wall and larger cell diameter.

### Insect wings

#### Cicada wing

The cicada species has recently attracted researchers’ attention because of their unique bactericidal wing properties. Cicadas live in a variety of environments: from underground to tall trees, high temperatures and humidity. Their wings allow them to adapt to different environments and consist mainly of chitin, protein and wax, covered with nano structures. Sun et al., characterized various nano-pillar geometries among 15 cicada species and found that nano-pillar diameter ranged from 82–148 nm, 44–177 nm pillar spacing and 159–146 nm in height [12, 44]. Nano-structure dimensions and the composition of the wax layer influence the hydrophobicity of the wing surface. Closely packed, highly ordered, tall nano-pillars show increased hydrophobic characteristics compared to disordered nano-pillar arrays [45]. The presence of the wax layer increases the contact angle of the nano-structures from a hydrophobic 76.8° to a superhydrophobic 146° contact angle [45, 46].

Ivanova et al. found that cicada wing surfaces kill *P. aeruginosa* cells within 3 min of contact [12]. This significant bactericidal ability motivates researches to focus on reproducing this structure on various substrates. Pogodin et al. presented a biophysical model of cicada nano-pillared surface interaction with bacterial cells. The model shows mechanical characteristics, particularly cell rigidity as important parameters in identifying bacterial resistance. Studies have shown that cicada wing surfaces have less of a bactericidal effect on gram-positive bacteria, due to their increased cell rigidity, compared to gram-negative cells [17].

#### Dragonfly wing

Dragonfly wings exhibit self-cleaning and bactericidal effects due to their superhydrophobic surface (153° contact angle) and distinct surface architecture [47]. The nano-structures found on the surface of dragonfly wings are primarily composed of aliphatic hydrocarbons, with fatty acids covering the outer most layer [48]. Rajendran et al. examined the wing membrane of dragonfly (*Sympetrum vulgatum*) wings using atomic force microscopy (AFM), identifying four main sections of nano-structures on the wing. These irregular shaped nano-structures were found to have dimensions varying between 83.3 and 195 nm [49]. A recent study demonstrated that the bactericidal efficacy of dragonfly wings were dependant on the nano-topology of protrusions on their wings [50]. Hence, different dragonfly species exhibit different degrees of bactericidal efficacy. While cicada wings are only efficient at killing gram-negative bacteria, dragonfly wings are capable of killing both gram-negative and gram-positive cells. At the current stage of research, it is unclear why this occurs [51], however a possible explanation is that the sharpness of the cicada wing nano-pillars are only able to pierce the thin gram-negative cell walls, but are insufficient for piercing thicker gram-positive cell walls [52].

#### Butterfly wing

Butterfly wings combine the anisotropic flow effects found on shark skin and the superhydrophobic properties of lotus and taro leaves to produce an effective anti-biofouling surface. Similar to lotus leaves, the surface of butterfly wings comprise of an array of aligned scales covered by hierarchal micro-grooves, approximately 1–2 µm in diameter [53, 54]. This structure produces a high contact angle (148°), allowing water droplets to roll off the surface of the wing in an axial manner, inducing self-cleaning. Aligned shingle-like scales on the wing, 30–50 µm in width and 58–146 µm in length cause this anisotropic behaviour. Anisotropic flow promotes low drag and water repellence, and this combined with superhydrophobic properties, results in a surface that has low drag, anti-biofouling and low bacterial adhesion properties [54, 55].

### Summary of natural surfaces

Table 1 summarises the information given in this section, presenting various natural surfaces, their individual surface textures and structure dimensions. It is important to note that these surfaces produce different behaviours. For example, the surfaces of plant leaves show anti-biofouling behaviour, which repel bacteria and impurities based on high contact angles (142°–159°). Surfaces such as cicada wings show bactericidal capabilities, with lower contact angles (76°–147°) than plant leaves. The large variation in structure dimensions and contact angles between the different species indicate that there is no one particular surface pattern that has universal antibacterial effects against all types of microorganisms.

**Fig. 1:**
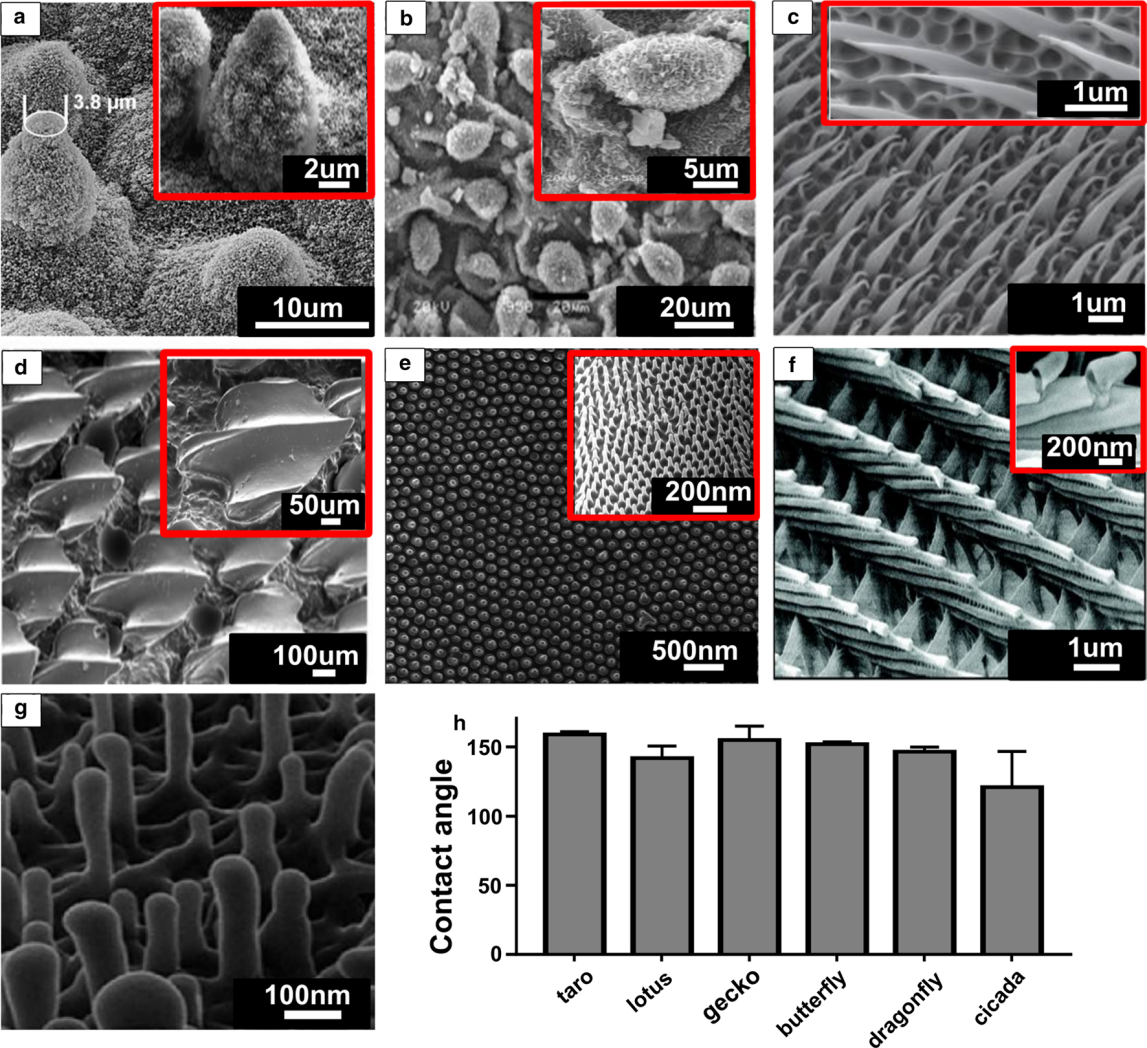
SEM images of nano-structured surfaces of: **a** lotus leaf [20, 146], **b** taro leaf [56], **c** gecko skin [147], **d** shark skin [148], **e** cicada wing [149], **f** butterfly wing [150] and **g** dragonfly wing [18]; **h** contact angles of naturally occurring bactericidal surfaces. Figures reproduced with permission

**Fig. 2:**
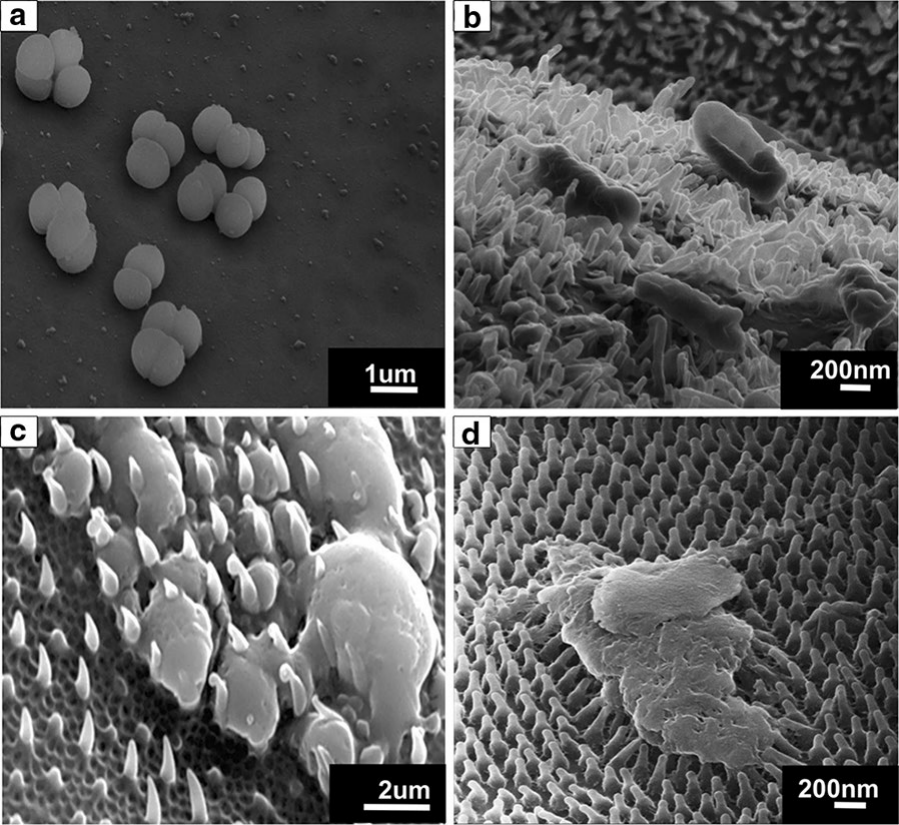
SEM images showing morphology of: **a** *S. aureus* on flat titanium [151], **b** *E. coli* on dragonfly wing [18], **c** *P. gingivalis* on gecko skin [43] and **d** *S. aureus* on cicada wing [149]. Figures reproduced with permission

Figure 1 shows scanning electron microscopy (SEM) images of the micro and nano-structures of various naturally occurring surfaces and their comparative contact angles. Figure 2 compares bacteria interacting with a flat titanium surface, cicada wing, dragonfly wing and gecko skin, showing the difference in antibacterial effects among the varying topographical structures. Figure 2a shows the bacteria cells undisturbed, with cell walls unchanged and adhering to the flat titanium surface, whereas disfiguration and piercing of the bacteria cells are observed in Fig. 2b–d.

## Artificial surface fabrication

The research focus on replicating naturally occurring surfaces has been a significant addition to the bioengineering field. A large number of studies have aimed to reproduce the antibacterial behaviour of certain naturally occurring surfaces, using a variety of chemical and mechanical methods. This section explores various methods of micro and nano-fabrication used to replicate this behaviour. Table 2 summarises the information in this section, explaining methods and techniques used, structures formed, and advantages and disadvantages of each method. Table 3 shows a summary of various fabricated surfaces, material of choice, bacteria strains tested and results obtained.

**Table 2:** Summary of nano and micro fabrication methods

Fabrication method		Structure dimensions	Advantages	Disadvantage	References
NIL		210 nm height nano-pillar	High throughput Low cost	Only applicable to polymers	[13, 15, 66]
UV-NIL		100 nm diameter nano-pillar	Lower deformation compared to NIL	Only applicable to cross-linkable polymers	[70]
Colloidal lithography		20 nm height pillars	Low consumption High throughput Easy to obtain colloidal crystals	Low resolution, often a secondary process is required to refine structures	[61]
Micro moulding		3D riblet of shark skin	Good resolution and high throughput at micro-scale	Not suitable for nano-scale structure Limited to polymers	[38, 43]
Vacuum casting		3D riblet of shark skin	Good resolution and high throughput at micro-scale Better resolution compared to micro moulding	Not suitable for nano-scale structure Limited to polymers	[39]
Femtosecond laser		20 µm elliptical structures with 200 nm nano-structures	Metal and non-metallic fabrication ability Good resolution and high throughput at micro-scale	Not suitable for nano-scale structure especially under the 200 nm	[75]
RIE		Pillar height 1.6 µm, with 350–750 nm diameter Pillar height 4 µm, 220 nm diameter	Good resolution Metal and non-metallic fabrication ability	High mask production costs	[81]
FIB milling					
Ga^+^-FIB		95 nm diameter pillars, length of 150–160 nm Nano holes 80–490 nm in diameter	High resolution Maskless Higher throughput compared to He+ Metal and non-metallic fabrication ability	Low throughput	[84, 102, 103]
He^+^-FIB		Sub 10 nm	High resolution Maskless Metal and non-metallic fabrication ability	Low throughput	[96, 104]
Hydrothermal synthesis		3 µm height pillars	Reliable Efficient Environmentally friendly Ability to control temperature and pressure	Reaction takes place in a sealed vessel, reducing throughput	[9, 14, 90]
Photolithography		Micro structure 1.5–20 µm	High throughput	Limited to photo sensitive material Low resolution	[63, 65, 95]
EBL		5–10 nm	High resolution High throughput	Only applicable to E-beam sensitive resists	[63, 96, 97, 100]

**Table 3:** Summary of studies investigating antibacterial effects of textured surfaces

Material	Fabrication method	Surface texture	Bio-inspiration	Bactericidal effects	References
Titanium	Hydrothermal etching	Nanowires	Dragonfly wings	Selective bactericidal activity (*P. aeruginosa* 50% cell death and *S. aureus* 20% cell death)	[14]
	Hydrothermal treatment	Nanostructured coating	–	Excellent bactericidal activity against *E. coli*	[88]
	Hydrothermal treatment	Nanowires	–	Bactericidal efficiency against *P. aeruginosa*, while increasing osteoblast and osteoclast cell growth. Optimised surface by varying reaction time	[91]
Titanium oxide	Hydrothermal method	Nanowires	Cicada wing	Selective bactericidal activity (*P. aeruginosa* > 50% cell death and *S. aureus* < 5% cell death)	[9]
Titanium oxide on silicon substrate	Glancing angle sputter deposition	Nanopillars	Cicada wing	Selective bactericidal activity (*E. coli* ~ 50% cell death and *S. aureus* no cell death) Did not affect adhesion of human mesenchymal stem cells and leukocytes	[105]
Gold on alumina template with silicon wafer	Electrodeposition	Nanopillars Nanorings Nanonuggets		Au nano-structures exhibited antibacterial properties, regardless of shape Number of live *S. aureus* cells on nano-structured surface was < 1% compared to flat surface	[106]
Silica alumina	Deep UV lithography and etching	Nanowells	–	Selective bactericidal activity based on cell morphology and surface topography (Circular, narrow rounded rectangular and wide rounded rectangular nanowell)	[60]
Silicon wafer	Urethane soft lithography	Nanopillars	Rice leaf and butterfly wing	Good drag reduction and self-cleaning properties	[54]
Black silicon	RIE and CVD	Nanopillars	Dragonfly wings	Excellent bactericidal activity against both gram-positive and gram-negative strains	[51]
Polyethylene terephthalate	Colloidal lithography	Nanocones	–	High aspect ratio of the nanocone arrays (up to 6) Promising anti-adhesion properties	[61]
Polymethyl methacrylate	Nil	Nanopillars	–	Moderate bactericidal effect against *E. coli* Smaller and more closely spaced nanopillars displayed better performance	[15]

Various types of lithography, such as deep ultraviolet (UV) lithography, electron beam lithography, X-ray lithography, colloidal lithography and nano-imprint lithography (NIL), are used to fabricate nano-structured surfaces [21, 60]. Lithography involves copying information or surface patterns from a master and transferring it to another surface. Some forms of lithography such as electron beam lithography (EBL) and scanning probe microscopy lithography, are time-consuming and costly for large-scale nano-structure fabrication [61]. Currently, colloidal and NIL are the most commonly used lithography methods for nanoparticle fabrication.

### Soft lithography

Soft lithography is an advanced polymer replication method, generally used for transferring micro and nano-structures onto polymer substrates. This technique involves a combination of printing, moulding and embossing with stamps [62]. Soft lithography is less expensive than other forms of lithography, as the fabricated mould can be re-used and does not require expensive processing [63]. It is an effective method for nanofabrication and when combined with etching, nano-structures can be transferred to metals for biosensing applications [62]. Soft lithography has been used by Wang et al. to fabricate bio-inspired pollen-like hierarchical surface structures. This surface is able to capture target cancer cells with high efficiency (72 ± 1.5%) and specificity. In this study, a negative replication of the pollen layer was formed using polydimethylsiloxane (PDMS). The viscous PDMS was poured onto the pollen layer and heated to cross-link and solidify the polymer [64]. While this method is effective, its use is limited to “soft matter”, such as organic and polymeric materials [62] and may therefore be inapplicable for fabricating large-scale nano-textured surface patterns on orthopaedic implants. While patterns can be transferred onto metal substrates, additional process are needed to do so.

### Nano-imprint lithography (NIL)

NIL, also known as hot embossing, is a contact form of lithography, which uses a mould to duplicate specified nano-structures onto a substrate surface. A layer of liquid polymer known as a “resist”, is placed onto the substrate surface and mechanically pressed with a fabricated stamp, leaving an imprint of the mould pattern in the substrate. The stamp is usually prepared using inorganic substrates, such as silicon [65]. Once the mould is removed the substrate may undergo reactive ion etching (RIE) to remove any residual resist and expose the substrate [66]. Dickson et al. reproduced the nano-structure pattern of cicada wings on a thin layer of poly methyl methacrylate (PMMA) using NIL. In this particular study, cicada wings were used as stamps to imprint their pattern onto the PMMA substrate. The study found that there was reduced adhesion of *E. coli* cells to the pillared surface compared to a flat surface [15]. NIL has also been used to produce nano-structures on indium phosphate, gallium phosphate and silicon substrates [13, 67], and to prepare micro-pillar patterned surfaces, inspired by gecko setae [68].

Compared to other methods of lithography, NIL has a high throughput rate, high resolution, rapid fabrication times and low cost. It combines multiple lithography and etching steps into one direct imprinting step, thereby reducing equipment and fabrication costs [65]. In addition, moulds can be re-used, further reducing the overall cost of the process. The biggest advantage of NIL over other forms of lithography, is that sub 2 nm patterning is achievable. Drawbacks of NIL include the limited pattern size, cost of mould fabrication, possible mould damage and the relative newness of the process, meaning that is not widely used [13, 66]. In addition, the removal of the mould from the target material causes damage to the structures [65]. Some researchers have used a UV-NIL process, in which UV radiation is used to cross-link polymeric nano-structures without structural deformation [69]. Cho et al. reproduced the nano-structure of dragonfly (*P. flavescens*) wings on glass, silicone, polyethylene terephthalate (PET) film, and curved acrylic polymers using UV-NIL [70].

### Colloidal lithography

Colloidal lithography uses colloidal crystals as a mask on the surface of a substrate. Several methods are used to form these crystals including vertical deposition, dip-coating, spin-coating and nano-robotic manipulation [71]. The crystals are arranged as a monolayer on the surface of the substrate and vapour deposition of the target material is initiated. Vapour reaches the substrate in the regions between crystals, leaving a pyramidal structure on the substrate. Upon vapour condensation, initial RIE processes remove the colloidal mask and further RIE processing increases nano-structure sharpness and refinement. This process may also involve additional steps, such as nano-lithography [21]. This technique has fabricated PET nanocone arrays to mimic bioinspired surfaces, and is used to form nano-patterned templates for biotechnological and biomedical applications [61, 72].

Colloidal lithography involves low consumption and high throughput, making it suitable for large-scale production. Colloidal crystals are generally easy to obtain and nano-structure dimensions are controlled through modulating the morphology of the colloidal mask and etching parameters, with longer etching times producing sharper nano-structures [61, 65]. This method is not without its drawbacks, however. The major issues facing colloidal lithography as a method of bio-mimicking are that the colloidal spheres limit pattern symmetry and the assembling process causes unavoidable defects [65]. While colloidal lithography has emerged as a new way of nano-fabrication for biomaterial applications, the process still needs to be optimised to reduce defects.

### Micro moulding

Micro moulding is a fast fabrication method for transferring nano-structures on to polymer substrates. In this process, the nano-pattern is filled with PDMS and the mould is replicated using epoxy resin. The original pattern is then removed, leaving a potential biomimetic surface replica [38]. This process is often used to reproduce the micro-structure of shark skin on epoxy resin. In a comparative study between micro moulding and NIL methods, the dimensional accuracy and degree of reproducibility of micro moulding was found to be higher than NIL. However, NIL provided higher fabrication accuracy on the outer edges of the substrate and on fine structures [38].

Hybrid methods of micro moulding combined with flame treatment has reproduced lotus leaf and shark wing patterns. In this case, the shark skin surface is first replicated via micro moulding, followed by a flame treatment to generate nano and micro-patterns which mimic lotus leaf structures. Nano and micro-structures formed by this method is highly dependent on the duration of flame treatment [73].

Li et al. replicated gecko skin structures using micro moulding, in which poly-vinyl siloxane (PVS) was used as the negative mould and epoxy resin for filling [43]. The majority of dimensions of the fabricated structures were close to that of natural gecko skin in terms of structure density, thickness and spacing. However, hair length and cap thickness were found to be largely different to natural structures [43]. Similarly, Zhang et al. found that there was significant replication error when reproducing the surface pattern of shark skin riblets using this method. This could be due to high pressures used during the process, causing bending and shrinkage of the natural surface, preventing high resolution replication [74].

### Vacuum casting

Vacuum casting is a common method used for replicating natural surfaces onto polymer and silicon substrates. In this process, a mould is put onto a PDMS substrate and is completely covered in unsaturated polyester resin containing glass fibres (used to eliminate cracking in the mould), under vacuum conditions. The mould is then removed from the resin and silicon is used to fill the space of the original mould under vacuum. The silicon is removed and the replication is left [39]. This method is commonly used to reproduce the pattern of shark skin. Similar to micro moulding, errors in replicating structure dimensions are attributed to the shrinkage of the mould during the process [39].

### Femtosecond laser

Femtosecond lasers fabricate superhydrophobic structures on various steels such as stainless steel, high speed steel and mould steel. This method mimicked the micro and nano-structured surface pattern of lotus leaves on titanium substrates, achieving specified dimensions. Fabricated structures were elliptical in shape, 10–20 µm in height, covered in 200 nm nanostructures, with a resulting in a contact angle of 144°. Colonisation of *S. aureus* was evident on this fabricated surface, while *P. aeruginosa* did not adhere to the surface [75]. A later study showed *S. aureus* cells adhered mainly in the crevices between micro-structures, which provided the cells better protection and less contact with the lotus-like titanium structures [76, 77]. Similarly, Epperlein et al. produced 700 nm homogeneous structures on corrosive and non-corrosive steel using Femtosecond laser production. Bacteria testing on these nano-structures showed clear antibacterial effects of the non-corrosive steel against *E. coli*. However, *S. aureus* cells were able to colonize on the same surface [78]. In comparison with this study, *S. aureus* adhesion was reduced when tested on a titanium nano-structured surface fabricated via Femtosecond laser processing. Structures were 750 ± 130 nm in diameter, 175 ± 40 nm in height and had a significant impact against biofilm formation [79]. Comparing these studies it is clear that nano-structure dimensions play a role in the antibacterial efficiency of the surface and that the Femtosecond laser process is a promising method for imparting antibacterial properties onto orthopaedic implants.

### Reactive ion etching (RIE)

RIE is a micro and nano-etching method using plasma to create nano-structures. High energy ions, generated by plasma under vacuum conditions, are bombarded onto the material surface causing localised material removal, forming nano-structure patterns [80]. RIE coupled with microwave plasma chemical vapour deposition (MPCVD) has replicated the nano-pattern of cicada wings on a diamond surfaces. Average structure heights were recorded to be 1.6 µm, with 350–750 nm widths [81]. Some studies have also used RIE to mimic the structured surface of dragonfly wings onto black silica or silicon wafers [51, 52].

### Focused ion beam (FIB) milling

FIB processing is similar to that of SEM processes, except that FIB deploys a beam of ions rather than electrons. FIB is effective in precisely milling nano-scale patterns, by selecting appropriate amounts of energy and intensity of the ion beam. A highly focused beam of Ga^+^ ions is applied at high beam currents, initiating the milling process. Gallium is currently the most commonly used ion source for FIB instruments for a several reasons including low vapour pressure, unique mechanical, electrical and vacuum features [82].

Nowadays, the FIB process is used in biomedical applications to image and analyse cells, and mill biomaterials [83]. FIB milling is an appropriate method for fabricating nano-structures (e.g. nanotubes) and nano-patterns for a variety of applications such as solar cells and fabricating nano-pillared semiconductor materials (95 nm diameter, 150–160 nm length) [84, 85].

### Hydrothermal synthesis

The term ‘hydrothermal’ refers to a heterogeneous reaction, in the presence of aqueous solvents under high temperature and pressure, which dissolves and recrystallises materials [86]. The process takes place in an autoclave vessel, where temperatures and/or pressures are controlled [87]. The hydrothermal process has produced a number of nano-structures, such as nanoparticles, nanorods, nanowires and nanotubes. Adjusting precursor concentrations, solvent composition, solvent pH, operation temperature and reaction duration, alters nanoparticle shape, size and surface roughness [87–89]. Researchers have employed this method to fabricate homogeneous spike-like structures on titanium to create micro-patterned arrays, inspired by the surface pattern of dragonfly wings [9, 14], as well as to test the influence of surface modification on bacterial adhesion in titanium-based materials [88].

Conventional hydrothermal processing produces micro-scale structures of spike height around 3 µm. Secondary processes, such as etching, has refined structures to a nano-meter scale [14]. Zhu et al. utilised supercritical hydrothermal conditions (400 °C) to fabricate TiO_2_ nanotubes with controlled morphology [90]. Reports on biological effects of nano-textured surfaces fabricated via this method have indicated a 50 and 25% inhibition of *P. aeruginosa* and *S. aureus* respectively, along with improved osseointegration, cell adherence and proliferation of fibroblast cells [14]. Tsimbouri et al. has used hydrothermal synthesis to fabricate titania nanowires, producing a surface that is bactericidal towards *P. aeruginosa* cells, while simultaneously promoting osteoblast and osteoclast growth [91]. This process is widely used for nanofabrication due to its reliability, efficiency, environmentally friendly nature and ability to control temperature and pressure during the process [14, 90].

### Sol–gel

The sol–gel method imparts favourable properties such as superhydrophobicity, onto metallic surfaces [4, 21]. In this process, hydrolysis and polymerisation reactions of precursors, such as inorganic metal salts or metal organic compounds, form a colloidal suspension called a sol. The gel forms as the sol is cast into a mould. The gel dries and goes through further heat treatment, converting it into ceramic material [87]. Heat treatments then improve the desired mechanical properties of the material. Nano-structure features are altered by parameters such as pH, amines, calcination temperature, and anodic membranes. For example, operating at a pH above 11 changes the structure shape from cuboidal to ellipsoidal. Desired nanoparticle size, crystal phase, and shapes, can be achieved through the sol–gel method [87].

The sol–gel method produces TiO_2_, by hydrolysis of alkoxide precursors and subsequent condensation of hydrolysed particles, forming a gel. The sol is prepared using titanium isopropoxide, and tetra-*n*-butyl-orthotitanate [5]. The sol–gel method is generally used as a part of a larger nano-fabrication process. For example, the sol–gel method is used to prepare seed layers for the controlled growth of nanoparticles during hydrothermal synthesis [92].

### Chemical and vapour deposition

Chemical vapour deposition (CVD) and physical vapour deposition (PVD) are not used as stand-alone nano-fabrication processes, but are widely used in coating and material property improvement. Both processes involve the deposition and condensation of evaporated target material on the surface of a substrate. CVD involves a chemical reaction in the vacuum chamber, where PVD does not [87].

Sputtering (e.g. magnetron sputtering and FIB sputtering) is a commonly used application of PVD. In the sputtering process, ions bombard a material surface causing local removal of substrate material ions from the surface. Magnetron sputtering is a well established, fluid-free process mainly used to deposit photocatalytic materials [93]. Nano-structures have been coated using magnetron sputtering in a study conducted by Huang et al. where twin gun reactive magnetron sputtering coated ZrO_2_, and ZrO_2_ doped with silver on titanium substrates [4, 94], as well as to coat TiO_2_ nano-dots with noble metals [93].

### Photolithography

Photolithography is one of the most popular methods of nano-scale fabrication [65]. The photolithography process begins with surface cleaning followed by coating a photoresist layer on the substrate, via spin coating. Positive and negative photoresists are used. Positive photoresists change chemical structure and become soluble when exposed to light, whereas exposure to light of a negative photoresist results in insolubility through polymerisation. A baking process strengthens the resist, enhancing adhesion of the resist to the substrate [63]. Patterns are transferred from the photolithography mask to the photoresist via UV light [65].

The mask is usually composed of a thin layer of chromium coated on a quartz or glass plates, is set on the photoresist layer and exposed to light. Soluble sections of the photoresist are removed using a developer solution, followed by etching, which affects areas not covered by the photoresist [63]. Although photomasks are easily available, there are significant costs and time involved in mask fabrication. In addition, surface chemistry is very difficult to control, and this method cannot be applied to curved surfaces [62]. While photolithography is widely used in the semiconductor industry, its viability is limited in biological applications. Negative photolithography needs a photo-cross-linkable polymer, however biocompatible polymers with photo-cross-linking ability are uncommon [95].

### Electron beam lithography (EBL)

EBL is the dominant method for producing nano-sized structures due to its lower proximity effect, high resolution and rapid throughput [96]. In the EBL process, the electron beam either images a surface or fabricates a resist previously deposited on a substrate. Due to the low energy of electrons, polymers such as PMMA, polyethylene glycol (PEG) and polyacrylic acid (PAA) are used as resist layers. Electrons produce negative lithography by cross-linking and positive lithography by degradation, depending on the type of mask. EBL can fabricate various feature dimensions (5–10 nm) [63, 96, 97], with the resolution of the structures depending on the molecule size of the resist, scattering range and backscattered secondary electrons [63]. EBL is able to fabricate much smaller structures than other methods of fabrication, such as photolithography [62]. Most biological applications of EBL have turned to biomolecule patterning to improve the functionality of polymers, with a large research focus on improving the absorption performances of biomolecules and self-assembling patterned protein monolayers [97–101]. While EBL is highly effective at producing high resolution and ordered patterns, the process involves the use of complex equipment, can only cover a small sample area and can be highly time consuming [65].

### Summary of artificial surface fabrication

Table 2 shows various methods of micro and nano-fabrication and compares their advantages and disadvantages. Replication methods such as NIL, micro moulding and vacuum casting have higher throughput, but are limited to soft materials such as polymers. RIE is an efficient method of fabricating nano-structures, but in order to fabricate precise structures mask preparation is needed, increasing costs. FIB milling is effective for high-resolution nano-fabrication in a micro-scale area. Hydrothermal synthesis is also effective and has been used in many studies involving bio-mimicking natural surface structures, due to its reliable and efficient nature.

**Fig. 3:**
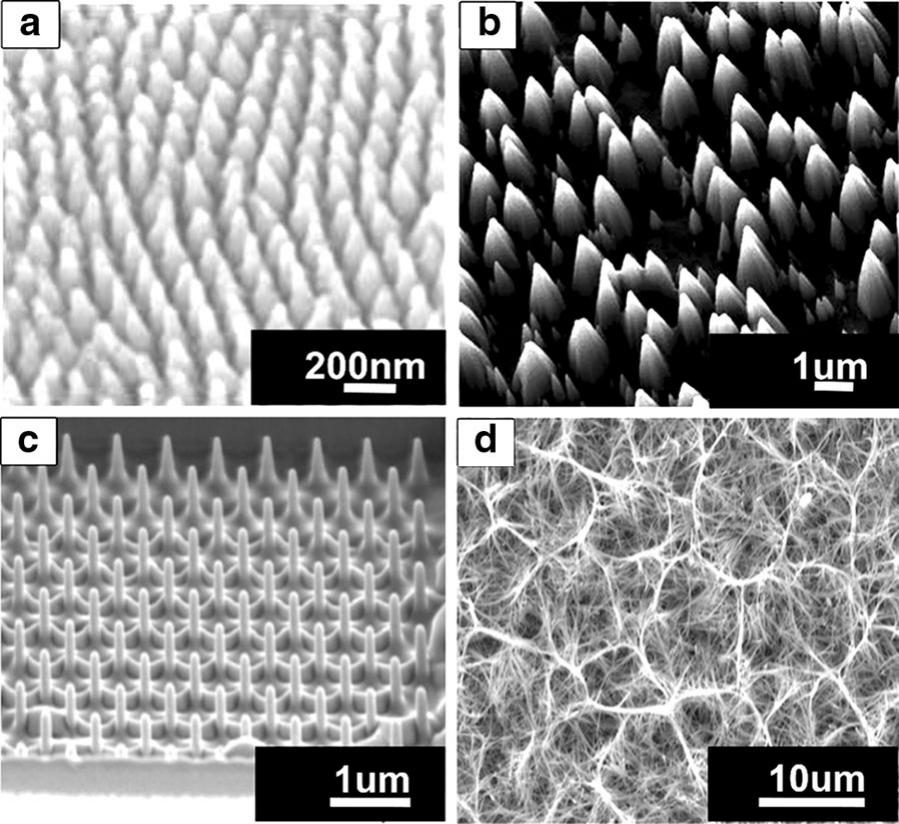
SEM images of structures fabricated via: **a** NIL [13], **b** RIE [81], **c** FIB milling [84] and **d** hydrothermal synthesis [9]. Figures reproduced with permission

Figure 3 shows SEM images of micro and nano-structures fabricated through four methods mentioned in this section. The images show that certain methods, such as FIB milling, allow for control over morphology, structure size and consistency, whereas hydrothermal synthesis produces randomly orientated and sized structures.

As illustrated in Table 3, according to recent studies bio-mimicked nano-structures of dragonfly using RIE and hydrothermal synthesis produce more effective bactericidal surfaces than other methods. The majority of studies found success in killing gram-negative bacteria cells, but not gram-positive bacteria. This is attributed to the thick, multilayered peptidoglycan structure that forms the cell wall of gram-positive bacteria. In addition, gram-positive bacteria generally shows a higher resistance to physical disruption than gram-negative bacteria, which explains the variation in results observed when both bacteria types are exposed to textured surfaces. The studies and methods mentioned in Table 3 have all had reasonable success in producing bactericidal, anti-biofouling or superhydrophobic surfaces.

## Bactericidal mechanism of nano-textured surfaces

Antibacterial surfaces often inhibit or lessen the growth of microorganisms either by their surface topography or by chemical modifications. Interestingly, the factors affecting bactericidal efficiency for different bacteria strains are different. Forces including van der Waals, Brownian motion, and electrostatic and hydrophobic interactions dictate reversible adhesion. Irreversible adhesion is more complex and involves cell characteristics and surface structure considerations [107]. Several innovative approaches have been employed to understand the mechanism involving bacterial death, with early models for bacterial adhesion proposed as early as 1971 [108]. These models however, tend to poorly correlate to experimental results due to the exclusion of factors such as hydrophobicity [107]. Surface wettability measurements is a key parameter used to assess the potential antibacterial behaviour of a surface. Materials with superhydrophobic surfaces (contact angle > 150°) have been found to prevent or reduce adhesion of bone marrow derived cells [109] and bacterial strains such as *S. aureus* and *P. aeruginosa* [110]. Many research groups have designed antimicrobial surfaces based on this cellular repulsion phenomenon, exhibited by natural surfaces such as taro and lotus leaves [56, 61, 111, 112]. However, the mechanism of microbial repulsion on superhydrophobic surfaces is complex and sparingly understood at this stage, as most gram-negative microbes have shown super repulsive nature, while gram-positive microbes tend to adhere onto these surfaces. More recent studies have shown a paradigm shift towards nano-textured surfaces where cell death is primarily caused by microbial membrane rupture via cellular adhesion.

To date, researchers have developed two models that explain the mechanism of prokaryotic microbial death on nano-patterned surfaces: (1) a biophysical model and (2) analytical thermodynamic model. The biophysical model demonstrates the interaction of prokaryotic microbes with superhydrophobic nano-pillar structures [17]. In this model, the bacterial cell membrane is considered as a thin elastic layer (neglecting details relating to structure and composition), due to the higher magnitude of thickness of the cicada wing nano-structure, compared to the bacterial membrane [17]. The main drawbacks of the two proposed mechanisms are that biological factors (e.g. bacteria composition, shape and structure) and mechanical properties of the nano-structures have been neglected. When bacterial strains such as *P. aeruginosa* or *P. claripennis*, adhere to the nano-pillars of cicada wings, the adsorbed layer is separated into two regions: a region where it is in direct contact with the pillar, and where it is suspended between pillars. This occurs because most bacterial cells are in the micro-meter range, while the textured surfaces are in the nano-meter range. The surface area of the region of direct pillar contact increases, stretching the cell membrane in the regions suspended between the pillars, leading to membrane rupture. Hence, according to this model, cell death is very much dependant on the rigidity of the bacterial cell membranes [17]. This may be the reason why rigid gram-positive bacteria strains are resistant to nano-patterned surfaces of cicada wings, when compared to less rigid gram-negative bacteria strains [11, 51]. Similar observations were also seen on fabricated nano-structures that resemble cicada wings [11].

**Fig. 4:**
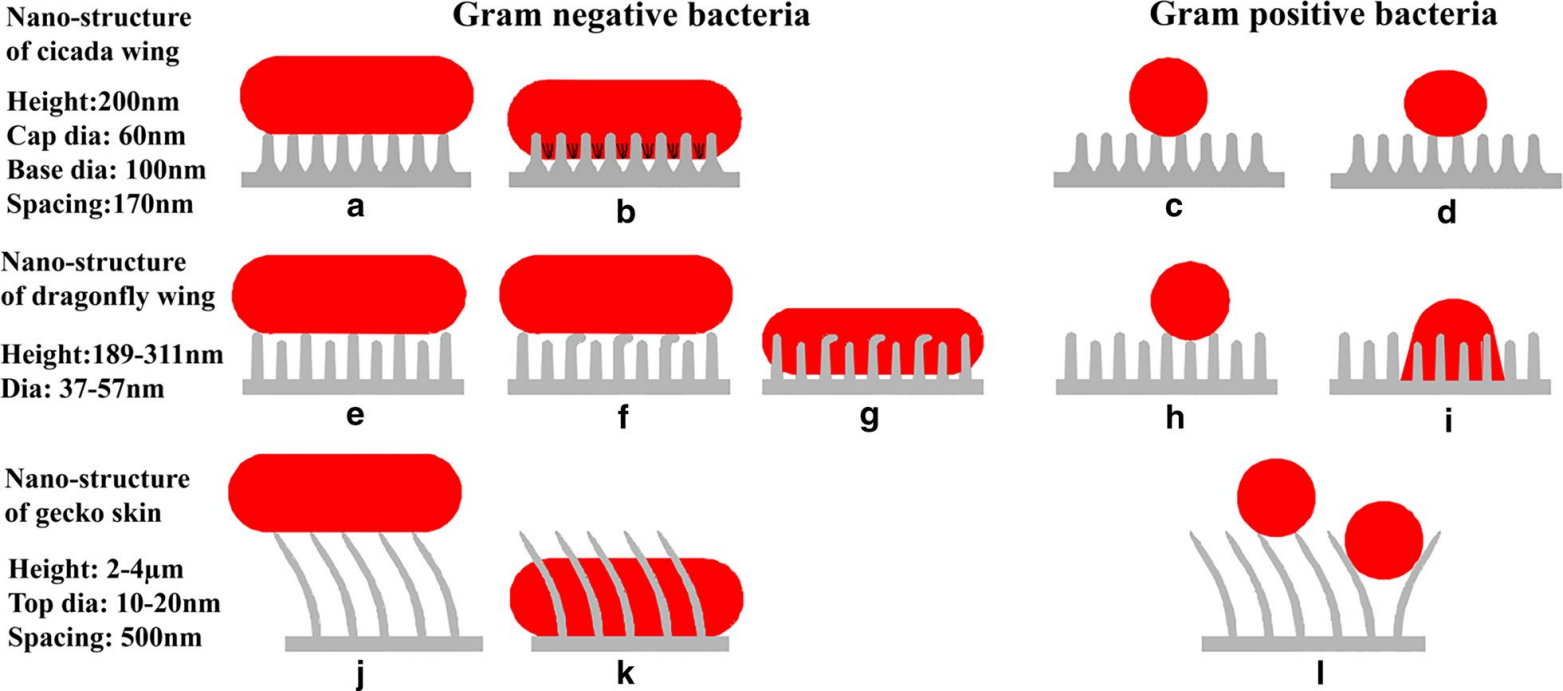
Schematic showing bacteria-nano-structured surface interaction of: **a**, **b** cicada wing and gram-negative bacteria, **c**, **d** cicada wing and gram-positive bacteria, **e**–**g** dragonfly wing and gram-negative bacteria, **h**, **i** dragonfly wing and gram-positive bacteria, **j**, **k** gecko skin and gram-negative bacteria and **i** gecko skin and gram-positive bacteria. Nano-structure dimensions are indicated next to each species, dimensions not to scale

Figure 4 demonstrates a schematic of the interaction of different nano-structure geometries with gram-negative and gram-positive bacteria. The mechanism of cell death of gram-negative bacteria by the cicada wing nano-structure is based on cell rupture, and normally occurs between the regions of space between nano-pillars (Fig. 4a, b) [12], while gram-positive bacteria resist this effect and live (Fig. 4c, d) [11]. When gram-negative bacteria, such as *E. coli*, is exposed to the nano-structure of dragonfly wings (pillar height 189–113 nm, and diameter 37–57 nm), taller nano-structures start to bend. Bacteria cells then strongly attach to nano-structures, due to the secretion of an extracellular polymeric substance (EPS) layer. When the adhesion force is strong enough, the bacteria membrane separates, due to the effort generated by the cell to move away from the nano-structure (Fig. 4e–g) [18]. The nano-structure of dragonfly wings also has bactericidal effects against gram-positive bacteria, such as *S. aureus*. Gram-negative bacteria, such as *P. gingivalis*, with diameter more than 500 nm, is penetrated by the nano-structure of gecko skin (Fig. 4j, k), while gram-positive bacteria, such as *S. mutans*, with a smaller diameter (< 300 to 400 nm) remain undamaged on top of the nano-structure [43].

A few years after this biophysical model was proposed, Li developed an analytic thermodynamic model, where the bactericidal mechanism of nano-patterned surfaces were interpreted via analysing the total free energy change of bacterial cells adhering to the patterned surface [23]. In this model, the stretching degree of the bacterial membrane is obtained from calculating the free energy change, when it is exposed to both flat and nano-patterned surfaces. The presence of nano-pillars increases the contact adhesion area, which increases the stretching degree of the membrane, leading to membrane rupture and death. A mathematical model developed to explain the mechanism of the bactericidal properties of cicada wings also utilises this “stretching” theory [113]. According to this model, maximum stretching of the bacterial layer is at the top of the nano-pillar ridges. Since gram-negative bacteria walls have fewer layers of peptidoglycans (1–3 layers) compared to gram-positive cells (10–50 layers), the maximum membrane stretching capacity of gram-negative bacterium is higher, leading to enhanced cell death. Cell-substrate adhesion strength has also been taken into account in determining the antimicrobial efficiency of nano-textured surfaces. A recent study revealed that nano-textured surfaces with high height to width aspect ratio displayed increased eukaryotic cell affinity than surfaces with lower aspect ratio. The surfaces which exhibited increased adhesion strength resulted in increased cell death [114]. Interestingly, dragonfly wings displayed a higher height to width aspect ratio than cicada wings, which may be the reason why dragonfly wings, and fabricated nano-textures resembling dragonfly wings, exhibit an increased antibacterial efficiency compared to cicada wings, and fabricated nano-textures resembling cicada wings [14, 50, 51, 105]. With recent developments in characterisation techniques, such as FIB milling, SEM and AFM, researchers have developed new insights into nano-textures and their properties, which have aided them in enhancing the bactericidal efficiency of these structures, by simply increasing the surface roughness, surface distribution density, radius, or height of nano-pillars. The use of software such as Autodesk^®^ Maya^®^ has enabled researchers to study the bacterial cell-surface interactions with the aid of three-dimensional (3D) visualisations and computer-generated animations [115].

Similar to nano-textured surfaces, chemically modified surfaces also kill microorganisms through direct contact and is generally achieved either by functionalising the surface with antibacterial functional groups like N,N- dimethyldodecylammonium bromide, quinoline or ammonium groups, or by coating the surface with antibacterial agents such as ammonium salts, silver nanoparticles, TiO_2_ nanoparticles, alkylated or halogenated polymers [116–131]. However, the application of chemically modified surfaces is limited due to its toxic effects on human cells, tissues or organs [132, 133].

## Stability and toxicity of micro and nanomaterials

The use of nano-patterned biomaterial implants in the body comes with concerns over the mechanical stability of the structures and unintentional health impacts of metal oxides, leading to long term toxicity concerns and potential cellular damage [134–136]. If the mechanical stability of nano-structures and dissemination of bio-coatings cannot withstand the biological environment of the body, exposure to metal oxides may cause interference to cells and organ function. Hence, establishing stability and cytotoxicity behaviour of such materials/implants are of vital importance. While the use of nanoparticle coatings are prevalent, dissolution of coating ions into the biological environment and loss of functionality over time is possible [137–140], which has large repercussions involving toxicity to the human body.

Sodium nitrate (Na_2_Ti_3_O_7_) nanowires fabricated by hydrothermal synthesis, exhibit brittle fracture behaviour upon bending, with non-linear elastic deformation observed. A single nanowire has an average Young’s modulus of 33 ± 7 GPa, with a yield strength of 2.7 ± 0.7 GPa [141]. At the current state of research in this field, the exact mechanical environment of the implant site is unspecified and hence, it is unknown whether the mechanical stability of the nanowires found is adequate to withstand the environment of the body. If individual nano-structures fracture in vivo, there may be associated toxicity effects. Since the toxicity of nano-structures is an unexplored research area, the toxicity of metal oxide nanoparticles can be considered as an initial judgement of toxicity.

“Needle-like” TiO_2_, Fe_3_O_4_, Al_2_O_3_, MoO_3_ and CrO_3_ nanoparticles have shown no effect on cellular shrinkage, and liver cells (in vitro) at low concentrations (10–50 µg/mL), however there is a significant effect at concentrations above 100 µg/mL [136, 142]. ZnO nanoparticles have caused cellular shrinkage and significantly decreased mitochondrial functionality at doses between 50 and 100 µg/mL, in a concentration, size and time dependent manner [136, 143]. CuO and Al_2_O_3_ nanoparticle exposure has been found to cause oxidative stress, with TiO_2_ nanoparticles causing liver damage in rats [144].

Silver (Ag) nanoparticles have been found to be toxic to mammalian cells derived from the skin, liver, lunch, brain, vascular system and reproductive organs, despite their excellent antimicrobial properties against *E. coli*, *S. aureus* and *Enterococcus faecalis* [123, 125, 132, 142, 145]. Similarly, Fe_3_O_4_ nanoparticle toxicity can cause inflammation and altered mitochondrial function, however their toxicity has so far shown no effect on liver cells in vitro, at low concentrations (100–200 µg/mL) [135, 136]. Factors such as environmental pH, nanoparticle aggregation, and average particle size, influence the degradation process of Fe_3_O_4_ in simulated body fluids. The stability of these particles also depended on the coating method, with coated particles showing slower degradation than uncoated particles [135].

The toxic nature of some of these metal oxides has shown their limited potential use in the human body. Micro and nano-structure fracture behaviour and mechanisms needs to be investigated to establish whether its presence will pose a risk to the body. If nano-structures were to fracture in vivo, short and long-term effects of the material fragments must be known. Hence, it is critical to determine the long-term mechanical stability and toxicity effects of nano-patterned surfaces and micro and nano-structures before they are deemed suitable for medical applications.

## Conclusion and future perspectives

The insertion of medical implants into the body comes with an associated risk of bacterial infection. This can often lead to long hospital stays, high health care costs, revision surgery or even death. Patients are commonly required to take long-term antibiotics to reduce the need for these treatments; however, the increasing resistance of bacteria strains to antibiotics has caused concern. Researchers are now aiming to find ways of preventing bacterial infection without the use of antibiotics. Currently, several methods of coating and ion-implantation of nano-particles improve antibacterial properties, osseointegration and bone regrowth on medical implants; however, their long-term use is limited. This has lead researchers to study the micro and nano-textured surface structures of naturally occurring bactericidal and anti-fouling surfaces, in the hope of reproducing this behaviour on to orthopaedic implant surfaces. The success of this replication may provide an alternative method of bacterial infection control after implant surgery, without the use of long-term antibiotics.

This review has summarised various natural surface structures, and recent advances in fabrication methods that replicate such nano and/or micro-patterns. Certain insect wings, plant leaves and animal skin prevent bacterial adhesion, and in some cases kill bacteria upon contact. This review found that dimensions, shape and configuration of these structures vary widely between species. This, coupled with the numerous fabrication methods and substrates materials used to replicate this behaviour, and with their varying bactericidal efficiencies, indicate that there is no one particular micro or nano-pattern which prevents or kills all types of microorganisms. Clearly, size, width, spacing, tip sharpness and height to width ratio have a major role in determining the bactericidal efficiency of the surface. Hence, a major challenge is to engineer a universal surface pattern that incorporates the best features of various naturally occurring nano and micro-surfaces. Research and experimentation in this area should also be expanded to include a wider range of pathogens, bacteria strains, surface structure dimensions, osteoblast assays and simulated body fluids.

Integration of current knowledge and new technologies is a key factor in developing smart antibacterial surfaces for medical implants. Methods that are particularly effective in mimicking this behaviour are FIB milling and hydrothermal synthesis, which is currently used to find the optimal surface for bactericidal behaviour by varying hydrothermal process parameters. Hydrothermal synthesis is currently the most commonly used method to fabricate nano-textured surfaces for antibacterial applications, due to its inexpensive nature and relative simplicity compared to other methods of fabrication.

Current research in bactericidal mechanisms and models provide an excellent starting point in understanding the mechanisms and behaviour that drive the bactericidal effects of textured surfaces. However, these models do not consider specific mechanical and biological cell membrane properties. Additional biological parameters such as bacteria structure, type (gram-positive or gram-negative), adhesion force, bacteria dynamics and nano-structure mechanical properties, need to be explored further and taken into account.

Furthermore, there is a lack of understanding of the mechanical stability and fracture mechanisms of micro and nano-structures. While toxicity effects of certain ions (such as silver) in the body are well established, micro and nano-structures present a new area of research in terms of the mechanical behaviour of individual nano-structures structures and the overall mechanical strength of the textured material. If the mechanical strength of individual structures is inadequate, structure fracture may occur, causing fragments to break away from the surface. The potential short and long-term effects of these fractured particles in the body must be established, as well as any changes to bactericidal behaviour if the structures were to be removed from the surface.

While the future of micro and nano-biomaterials is exciting and promising, researchers have only just begun to scratch the surface of this field. While we currently have an excellent starting point, there is still a fair amount of research to be completed before the successful implementation of nano-textured orthopaedic implants. Large-scale, rapid production methods of uniform nano-structures remains difficult. In addition, researchers need to optimise the textured surface to inhibit bacteria adhesion and growth against both gram-negative and gram-positive bacteria strains, while simultaneously promoting osteoblast metabolic activity and bone regrowth. Ideally, the production and insertion of textured bactericidal orthopaedic implants will lower the rate of implant failure due to bacterial infection. This potentially reduces post-surgery recovery and hospitalisation times, healthcare costs, revision surgery and death rates, and the need for long-term antibiotics.

## Abbreviations

*P. aeruginosa*: *Pseudomonas aeruginosa*
*S. aureus*: *Staphylococcus aureus*
*S. epidermidis*: *Staphylococcus epidermidis*
*S. mutans*: *Streptococcus mutans*
AFM: atomic force microscopy
UV: ultraviolet
NIL: nanoimprint lithography
RIE: reactive ion etching
PDMS: polydimethylsiloxane
PMMA: poly methyl methacrylates
PET: polyethylene terephthalate
PVS: poly-vinyl siloxane
MPCVD: microwave plasma chemical vapour deposition
FIB: focused ion beam
SEM: scanning electron microscopy
TEM: transmission electron microscopy
CVD: chemical vapour deposition
PVD: physical vapour deposition
EBL: electron beam lithography
PEG: polyethylene glycol
PAA: polyacrylic acid
